# Coordination Between ROS Regulatory Systems and Other Pathways Under Heat Stress and Pathogen Attack

**DOI:** 10.3389/fpls.2018.00490

**Published:** 2018-04-16

**Authors:** Nobuhiro Suzuki, Kazuma Katano

**Affiliations:** Department of Materials and Life Sciences, Faculty of Science and Technology, Sophia University, Tokyo, Japan

**Keywords:** abscisic acid (ABA), Ca^2+^ signaling, growth stages, programmed cell death (PCD), reactive oxygen species (ROS), respiratory burst oxidase homologues (RBOHs)

## Abstract

Regulatory systems of reactive oxygen species (ROS) are known to be integrated with other pathways involving Ca^2+^ signaling, protein kinases, hormones and programmed cell death (PCD) pathways to regulate defense mechanisms in plants. Coordination between ROS regulatory systems and other pathways needs to be flexibly modulated to finely tune the mechanisms underlying responses of different types of tissues to heat stress, biotic stresses and their combinations during different growth stages. Especially, modulation of the delicate balance between ROS-scavenging and producing systems in reproductive tissues could be essential, because ROS-dependent PCD is required for the proper fertilization, despite the necessity of ROS scavenging to prevent the damage on cells under heat stress and biotic stresses. In this review, we will update the recent findings associated with coordination between multiple pathways under heat stress, pathogen attack and their combinations. In addition, possible integrations between different signals function in different tissues via ROS-dependent long-distance signals will be proposed.

## Introduction

Plants are exposed to multiple abiotic and biotic stresses that may simultaneously occur in the natural environments. Although necessity of the researches deciphering the molecular mechanisms underlying response of plants to stress combinations has been proposed in many reviews, such studies are still scarce (Suzuki et al., 2014; Pandey et al., 2015). In addition, several transcriptome studies demonstrated that unique mechanisms that govern the responses of plants to stress combinations could not be easily predicted from the studies focusing on individual stress factors (Atkinson et al., 2013; Prasch and Sonnewald, 2013; Rasmussen et al., 2013). Under the natural environment, effects of pathogens on plants can be altered by abiotic factors (Pandey et al., 2015). Indeed, mechanisms that regulate defense responses in plants were shown to be different under different temperatures (Aoun et al., 2017). For example, heat stress, high temperature that negatively impacts plant growth, enhanced sensitivity of plants to pathogens in many cases (Pandey et al., 2015). In addition, the level of crosstalk between heat responses and defense responses might depend on several factors such as plant species, tissues, developmental stage, stress intensity, and timing (Nejat and Mantri, 2017).

A recent review proposed that strategies of plants to adapt to stress combinations consists of both shared and unique mechanisms, and shared mechanisms constitute a considerable portion of plants’ responses to both individual and combined stresses (Pandey et al., 2015). Reactive oxygen species (ROS) regulatory systems might be one of the most essential shared mechanisms to modulate the machineries governing defense and acclimatory responses to minimize the damage on cells (Suzuki et al., 2012; Rejeb et al., 2014; Pandey et al., 2015). Although ROS have long been known as harmful compounds under stress conditions, several lines of evidences indicated the significance of ROS as signaling molecules in the regulation of various biological processes (Choudhury et al., 2013; Mittler, 2017). ROS might activate the mechanisms that attenuate the effects of oxidative stress caused by abiotic stress, as well as programmed cell death (PCD) under biotic stresses (Kissoudis et al., 2014). Furthermore, ROS as universal signals might integrate with other pathways such as Ca^2+^ signaling, kinase cascades, and hormone signaling to tailor the specific mechanisms to adapt to different types of biotic and abiotic stresses (Mittler et al., 2011; Suzuki et al., 2013a).

Taken together, we can hypothesize that plants might possess the ability to flexibly coordinate the ROS regulatory systems and other integrating pathways to tailor the cellular homeostasis depending on the types of biotic and abiotic stresses individually or simultaneously occurred, and depending on the growth stages and types of tissues. In this review, we will not address detailed mechanisms underlying the response of plants to combinations of biotic and abiotic stresses, which have been discussed in previous reviews (Atkinson and Urwin, 2012; Kissoudis et al., 2014; Zhang and Sonnewald, 2017), but focus on coordination between ROS regulatory systems and other pathways under heat stress and pathogen infections. Especially, coordination of signals depending on different growth stages and tissues, and links between different signals function in different tissues will be suggested.

## Signaling Pathways Involved in Heat Response, Defense Response and Pcd in Different Growth Stages and Tissues

Pathways involved in heat responses have been extensively studied using seedlings and vegetative tissues. More than decade ago, Larkindale et al. (2005) analyzed heat tolerance in seedlings of Arabidopsis mutants deficient in different signals, and demonstrated the significance of various pathways such as ROS regulatory systems and hormone signaling including abscisic acid (ABA), salicylic acid (SA), jasmonic acid (JA) and ethylene signaling as well as heat shock protein (HSP)-dependent pathways in the heat response of plants. In addition, Arabidopsis plants deficient in *S*-nitrosoglutathione reductase (GSNOR), which metabolizes the nitric oxide (NO) adduct *S*-nitrosoglutathione, were more sensitive to heat stress compared with WT plants (Lee et al., 2008), suggesting the involvement of GSNOR-dependent NO metabolism in heat tolerance of plants. Recent studies using vegetative tissues of Arabidopsis plants identified the four putative heat sensors localized in different subcellular components. They include a calcium channel on the plasma membrane, a histone sensor in the nucleus, and two unfolded protein sensors in the endoplasmic reticulum (ER) and the cytosol (Sugio et al., 2009; Che et al., 2010; Kumar and Wigge, 2010; Finka et al., 2012). The deficiency in one of the putative heat sensors, cyclic nucleotide-gated channel 2 (CNGC2) resulted in enhanced heat tolerance in seedlings accompanied by enhanced accumulation of HSPs as well as increased cytosolic Ca^2+^ level (Finka et al., 2012). Furthermore, heat sensing mechanisms involving unfolded protein responses in the ER and the cytosol might be linked via ROS regulatory systems during vegetative stages (Sun and Guo, 2016; Kataoka et al., 2017). More recently, phytochrome B was shown to be another heat sensor that mediates the switching of cellular status between growth-promoting mode and heat-acclimation mode (Jung et al., 2016; Quint et al., 2016). Some of these pathways implicated in heat responses in seedlings and vegetative tissues were also shown to contribute to defense responses. For example, CNGC2, also known as defense no death 1 (DND1) is required for the activation of PCD under pathogen attack (Clough et al., 2000). In addition, phytochrome B was shown to regulate the defense pathway involving lipoxygenase and mitogen-activated protein kinases 3 and 6 (Zhao et al., 2014). Furthermore, involvement of key players of heat responses such as ROS regulatory systems, Ca^2+^ signaling, kinases and various hormones in defense responses has been demonstrated in previous studies (Zhang et al., 2016, 2017; Luo et al., 2017). For example, a lectin receptor-like kinase (LecRK-IX.2) was shown to be required for the defense mechanisms triggered by pathogen recognition receptors. LecRK-IX.2 might induce phosphorylation of a ROS producing NADPH oxidase, respiratory burst oxidase homologue D (RBOHD) by recruiting Ca^2+^-dependent protein kinases to trigger ROS production (Luo et al., 2017). In addition, overexpression of ethylene response factor 014 (ERF014) resulted in enhanced resistance of Arabidopsis to pathogens accompanied by enhanced expression of SA response genes and oxidative burst that was shown to be induced by RBOHD (Zhang et al., 2016), suggesting the roles of ERF014 in the regulation of SA signaling and ROS production in defense responses.

**FIGURE 1:**
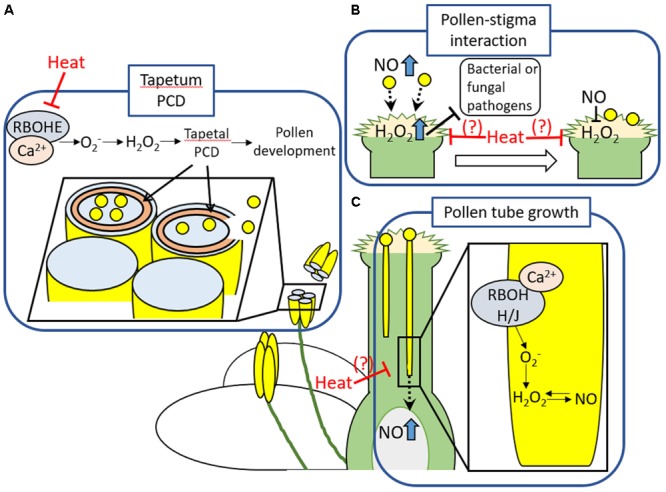
Developmental processes in reproductive tissues involving ROS production or PCD. **(A)** RBOHE that might be activated by Ca^2+^ produces ROS to enhance tapetal PCD. This process occurred in the appropriate timing might be required for the proper development of pollens. **(B)** High level of NO and H_2_O_2_ accumulation in pollens and papilla cells, respectively, might be essential for the recognition of pollens by papilla cells followed by the attachment of pollens to the top of stigma. In addition, level of H_2_O_2_ could be sufficient to protect papilla cells against pathogens, but, not as much as causing damage (Hiscock and Allen, 2008). Following the attachment of pollen on the stigma, NO derived from pollen might function to decrease the level of H_2_O_2_ accumulation in stigma. The same tendency in ROS and NO level at the arrival of pollens to pistils was commonly observed among the different species (Zafra et al., 2010). These findings suggest that dramatic shift of the ROS-NO redox state could be required for initiation of pollen germination. However, significance of the decrease in H_2_O_2_ level in papilla cells in fertilization is still not clear. **(C)** RBOHH and RBOHJ that might be activated by Ca^2+^ produce ROS to regulate pollen tube growth. Appropriate level of RBOHH/J-dependent ROS production might be maintained by NO signals. Production of NO in ovule might be also involved in guidance of pollen tube growth (Serrano et al., 2015). These developmental processes required for fertilizations are sensitive to heat stress. Indeed, tapetal PCD was shown to be inhibited by heat stress (De Storme and Geelen, 2014). Effects of heat stress on ROS signaling involved in other developmental processes in reproductive tissues should be elucidated in more detail.

High sensitivity of reproductive tissues especially male reproductive tissues to heat stress, has been addressed in many studies (Zinn et al., 2010; Giorno et al., 2013). It should be noted that processes regulating reproductive development and fertilization share common features with mechanisms underlying defense responses, such as increase in RBOH-dependent ROS production and cytosolic Ca^2+^ (Chen et al., 2015) (**Figure 1**). For example, appropriate timing of tapetal degeneration involving PCD regulated by RbohE-mediated proper temporal ROS regulation was shown to be essential in pollen maturation and fertility (Xie et al., 2014; Kurusu and Kuchitsu, 2017) (**Figure 1A**). In addition, ROS and NO also play an important role in pollen-pistil interactions, as well as pathogen defense (Hiscock and Allen, 2008; Traverso et al., 2013; Serrano et al., 2015) (**Figure 1B**). Prior to and during arrival of pollens to pistils, high level of NO and H_2_O_2_ in pollens and papilla cells, respectively, might be required for the recognition of pollens by papilla cells. Following the arrival of pollen grains on a tip of stigma, NO derived from pollens might function to decrease the level of H_2_O_2_ accumulation in papilla cells (Zafra et al., 2010; Traverso et al., 2013; Serrano et al., 2015). Furthermore, RBOHH- and RBOHJ-dependent ROS production integrated with Ca^2+^ and NO signaling might be required for the pollen tube elongation (Domingos et al., 2015) (**Figure 1C**). During this process, NO-ROS redox signal might navigate pollen tube growth to the proper direction (Prado et al., 2008; Traverso et al., 2013; Serrano et al., 2015). These processes implicated in reproductive development and fertilization were shown to be sensitive to heat stress (Zinn et al., 2010; De Storme and Geelen, 2014). Despite the significance of RBOHs-dependent ROS production and PCD in pollen development and fertilization, pollen PCD might be also one of the main cause of damages on plant reproduction during heat stress (De Storme and Geelen, 2014). Pollen and tapetal cells were known to possess great number of mitochondria, one of the main source of ROS in plant cells (Muller and Rieu, 2016). Imbalance between mitochondrial ROS production and countering scavenging pathways could lead to the dysregulation of PCD during heat stress (Muller and Rieu, 2016). These findings therefore indicate the significance of cellular ROS homeostasis in the protection of reproductive tissues against heat stress and abiotic stresses.

Heat stress can also alter carbohydrate distribution in source and sink tissues, and resulted in impaired production of seeds and fruits (Sato et al., 2006). This heat-induced imbalance in carbohydrate distribution could be further exacerbated by pathogens (Zhang and Sonnewald, 2017). Dysregulation of carbohydrate distribution under heat stress accompanied by pathogen infection could affect the mechanisms underlying the PCD, and probably ROS signaling, because tapetal PCD was also shown to be required for the delivery of carbohydrates and other compounds necessary for pollen development (Muller and Rieu, 2016). In addition, ABA that might switch positive and negative effects of abiotic stimuli on defense responses in plants (Rejeb et al., 2014), could be also involved in the regulation of carbohydrate distribution. A previous study demonstrated that sucrose non-fermenting-1-related protein kinase (SnRK1), a regulator of ABA signaling (Tsai and Gazzarrini, 2014; Zhang and Sonnewald, 2017) was shown to be required for sugar sensing (Li and Sheen, 2016) and communication between sink and source under various abiotic stresses (Lin et al., 2014).

Taken together these findings suggest that ROS regulatory systems and other pathways such as PCD pathways, Ca^2+^ signaling and hormone signaling needs to be strictly coordinated in plants depending on the different growth stages and tissues to finely tune the mechanisms underlying proper development, and responses to heat stress and pathogen attack.

## Possible Links Between Signals Activated in Different Tissues

ROS-dependent heat response mechanisms might be differently modulated in vegetative and reproductive tissues. For example, expression pattern of genes regulated by HSFA2 that might be involved in H_2_O_2_ sensing was shown to diverge between leaves and anthers under heat stress (Muller and Rieu, 2016). In addition, Arabidopsis plants deficient in a cytosolic ROS scavenging enzyme, ascorbate peroxidase 2 (APX2) showed enhanced heat sensitivity in seedlings, while enhanced heat tolerance in seed production (Suzuki et al., 2013b). Conversely, deficiency in CNGC2 in Arabidopsis resulted in enhanced heat tolerance in seedlings, but, enhanced heat sensitivity in seed production (Katano et al., 2017). The difference in heat tolerance between seedlings and reproductive tissues in plants deficient in CNGC2 might be due to the differences in ROS regulatory systems. These findings indicate that effects of heat stress on ROS-dependent pathogen defense could be also different depending on growth stages and tissues. Indeed, expression of CNGC2 was shown to be altered depending on the growth stages (Finka et al., 2012; Katano et al., 2017). Cross talk between CNGC2-dependent heat response and defense response can be also supported by the fact that CNGC2 is also known as DND1 that is involved in activation of PCD during pathogen attack (Clough et al., 2000). In addition, infection of tomato yellow leaf curl virus mitigated the heat response via inhibiting HSFA2 and APX2 (Anfoka et al., 2016).

**FIGURE 2:**
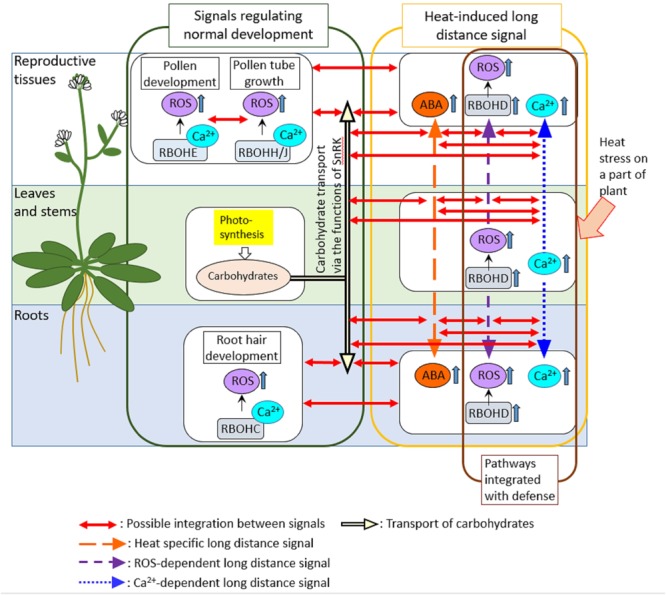
Possible integrations of signals activated in different tissues. In this review, we suggest the possible integration between different signals that regulate development, heat response and pathogen defense in different tissues via ROS-dependent long-distance signaling. Long-distance signals that can be activated by heat stress and pathogens might involve Ca^2+^ signals as well as RBOHD-dependent ROS signals. Therefore, other RBOH proteins that function in different tissues to regulate developmental processes could be activated by Ca^2+^ signals propagated from the different part of the plant. In addition, carbohydrate transport that might be regulated by ABA signals could be also integrated with ROS- and Ca^2+^-dependent long-distance signals. Indeed, ABA was shown to function together with RBOHD-dependent ROS signals to activate heat-induced long-distance signaling (Suzuki et al., 2013a). Furthermore, tapetal PCD was also shown to be required for the delivery of carbohydrates necessary for pollen development, suggesting the integration between PCD pathways and mechanisms that regulate carbohydrate distributions in plants.

We cannot ignore the possibility that long-distance signaling might link the mechanisms activated in different tissues (**Figure 2**). In Arabidopsis, each different RBOH protein might function in different tissues and play key roles in the regulation of different biological processes (Suzuki et al., 2011). For example, RbohE plays key roles in the regulation of PCD in tapetal cells that is required for proper development of pollens (Xie et al., 2014). On the other hand, RBOHC was shown to be required for development of root hairs (Knight, 2007). In addition, RBOHD is required for long-distance signaling that can be propagated through entire plant when part of a plant is subjected to pathogen attack or heat stress (Dubiella et al., 2013; Suzuki et al., 2013a). It should be interesting to address if RBOHD-dependent long-distance signals integrate with signals activated by other RBOHs. Integration of different RBOHs via long-distance signals could be also hypothesized by the fact that Ca^2+^ signaling which might be required for the activation of RBOH proteins is also propagated through entire plant in response to biotic or abiotic stresses on part of a plant (Gilroy et al., 2016). In addition, it might be also necessary to investigate how long-distance signaling and its integration with other pathways are coordinated in response to heat stress, pathogen attack and their combinations. A previous study provided a good example showing the activation of long-distance signaling by the local application of abiotic stress (high light), leading to the activation of defense mechanisms in the distal leaves (Karpinski et al., 2013).

It could be also interesting to study integration of long-distance signaling induced by heat stress or pathogens with carbon transport between sink and source tissues, because of the certain level of overlaps in the mechanisms between these processes (**Figure 2**). ROS-dependent signal might be integrated with heat-specific long-distance signal that might enhance ABA synthesis, but decrease the intermediate of sugar metabolism in leaves not directly exposed to heat stress when part of a plant was subjected to heat stress (Suzuki et al., 2013a). Ca^2+^ signaling that might integrate with ABA- and SnRK-dependent pathways (Wasilewska et al., 2008) was also shown to be integrated with ROS-dependent long-distance signaling in response to local application of abiotic and biotic stimuli (Gilroy et al., 2016). These findings suggest that the mechanisms to balance the assimilate use for plant development, and responses to abiotic and biotic stress rely on integration between several pathways, including ROS production, Ca^2+^ signaling, metabolite sensing and hormone balance.

## Cross Talk Between Ros-Dependent Signals and Other Pathways Under Combinations of Heat Stress and Pathogen Attack

Defense responses might be modulated depending on temperature. In many cases, abiotic stresses negatively impact on disease resistance of plants (Huot and Castroverde, 2017). For example, ornamental plant roots directly exposed to high soil temperatures increased severity of *Phytophthora infestans* (Pandey et al., 2015). The heat-dependent suppression of disease resistance might be due to the inhibition of hypersensitive response and R-gene mediated defense responses (Pandey et al., 2015; Huot and Castroverde, 2017). Nevertheless, several studies demonstrated the enhanced disease resistance by abiotic stresses. Enhancement of rust pathogen resistance of wheat regulated by Yr36 gene, which was accompanied higher accumulation of ROS and activated HR was observed especially under high temperature (Li et al., 2016).

Comprehensive expression analysis of HSPs in wheat revealed that different types of HSPs can be enhanced in response to different types of pathogens (Muthusamy et al., 2017). NO and ROS regulatory systems might be involved in the regulation of HSP- and HSF-dependent pathways during heat stress and pathogen infection. For example, compounds that produce or scavenge NO and an inhibitor of ROS producing NADPH oxidases affected HSP70 accumulation under heat stress and a combination of heat stress and pathogen infection (Piterkova et al., 2013). In addition, mitochondrial uncoupling protein in tomato (LeUCP) might also play key roles in the regulation of heat tolerance and pathogen defense (Chen et al., 2013). Overexpression of LeUCP resulted in enhanced tolerance of tomato plants to heat stress as well as *Botrytis cinerea* accompanied by lower accumulation of ROS. In this case, cross talk between heat response and defense response might be strictly regulated by fine-tuned mechanisms to balance between ROS scavenging to prevent oxidative damage caused by heat stress and ROS production necessary for the defense responses. This finding therefore suggests that mitochondrial ROS regulatory systems might play key roles in the ROS-dependent cross talks between heat response and pathogen defense. It should be also interesting how UCP-dependent mechanisms might modulate pathogen defense and PCD under heat stress in pollens that contain many mitochondria (Muller and Rieu, 2016).

A recent study demonstrated that Xa7-mediated pathogen resistance in rice might function better under high temperature (Cohen et al., 2017). This Xa7-mediated pathogen resistance might be regulated by inhibition of ABA signaling, but not by activation of SA signaling. Roles of ABA in the cross talk between biotic and abiotic stresses are still controversial. Although ABA plays key role in the stomatal closure a common entry point for pathogen (Lim et al., 2015), ABA might antagonistically interact with pathogen response involving SA signaling. This dual effect indicates the role of ABA as a switch to modulate positive and negative effects of heat stress on defense mechanisms (Rejeb et al., 2014). Furthermore, ABA was shown to play pivotal role to tailor the response of plants to various stress combinations via integration with other hormones and ROS regulatory systems (Suzuki, 2016).

## Conclusion

Based on the previous findings, we propose that coordination between ROS regulatory systems and other pathways such as Ca^2+^ signaling and hormone signaling should be finely tuned in plants under heat stress, pathogen attack and their combination. Such coordination between multiple pathways might be modulated depending on the growth stages and type of tissues. Although appropriate ROS scavenging is essential to prevent damage on cells under heat stress (Chen et al., 2013), ROS-dependent PCD is also essential to regulate pathogen defense in plants (Kissoudis et al., 2014). In this context of the balance between ROS scavenging and PCD, it might be urgent to elucidate the mechanisms underlying the response of reproductive tissues to heat stress, pathogen attack and their combinations, because PCD is essential for the appropriate development of pollens and fertilization (Kurusu and Kuchitsu, 2017).

ABA signaling and carbohydrate distributions that might be integrated with ROS regulatory systems and Ca^2+^ signaling are shown to be modulated through the entire plants over the long distance. Furthermore, different RBOH proteins were shown to be specifically expressed in different tissues. Therefore, it should be necessary to address how heat acclimation and defense mechanisms in different tissues are linked via long-distance signaling under heat stress, pathogen infection and their combinations.

## Author Contributions

All authors listed have made a substantial, direct and intellectual contribution to the work, and approved it for publication.

## Conflict of Interest Statement

The authors declare that the research was conducted in the absence of any commercial or financial relationships that could be construed as a potential conflict of interest.
